# Sex-, age-, and time-specific visual communication in brown bears

**DOI:** 10.1093/jmammal/gyac126

**Published:** 2023-02-09

**Authors:** Vincenzo Penteriani, Léa Etchart, Enrique González-Bernardo, Alfonso Hartasánchez, Daniele Falcinelli, Héctor Ruiz‑Villar, Ana Morales‑González, María del Mar Delgado

**Affiliations:** National Museum of Natural Sciences (MNCN), Department of Evolutionary Ecology, Spanish National Research Council (CSIC), c/José Gutiérrez Abascal 2, 28006 Madrid, Spain; UMR 6249 Chrono-environnement, Université de Bourgogne Franche-Comté, 16 route de Gray, 25000 Besançon, France; Departamento de Zoología, Universidad de Granada, Facultad de Ciencias, Avda. Fuente Nueva S/N, E-18071 Granada, Spain; FAPAS Fondo para la Protección de los Animales Salvajes, Ctra. AS-228, km 8,9 – Tuñón, 33115 Santo Adriano, Asturias, Spain; Department of Environmental Biology, Sapienza University of Rome, 00185 Rome, Italy; Biodiversity Research Institute (IMIB, CSIC-Oviedo University-Principality of Asturias), Mieres Campus, 33600 Mieres, Spain; Department of Conservation Biology, Estación Biológica de Doñana, C.S.I.C, Avda. Americo Vespucio 26, 41092 Seville, Spain; Biodiversity Research Institute (IMIB, CSIC-Oviedo University-Principality of Asturias), Mieres Campus, 33600 Mieres, Spain

**Keywords:** chemical marking, debarking, large carnivores, mating, rubbing trees, *Ursus arctos*, visual marking, visual signaling

## Abstract

Intraspecific communication in mammals is well-documented but generally restricted to chemical and acoustic signaling. However, other overlooked channels, such as visual signaling, may be used to communicate among conspecifics. Here, by using experimental manipulations together with camera traps on 13 brown bear (*Ursus arctos*) rubbing trees in the Cantabrian Mountains (northwestern Spain), we document detailed temporal patterns and behavioral aspects of a recently discovered novel communication channel for this species, visual signaling through the trunk debarking of focal trees. Video footage showed that visual marking is a sex-, age-, and time-specific means of communication in brown bears, being performed exclusively by adult males during the mating season (mainly April–June in the study area). Trunk debarking was always associated with chemical marking and was never an isolated behavior, suggesting that visual and chemical signals might be complementary. Visual and chemical marks may provide different information; for example, visual marks could be an indicator of individual size and, thus, the dominance status of adult males looking for mating opportunities. This is the first time that evidence is provided showing that visual signaling in a large carnivore is exclusive to a specific class of individuals (adult males) and linked to reproductive needs only. Bear visual signaling not only represents an advance in our comprehension of animal communication but may also serve to easily locate the mating areas of mammals, which are crucial for large carnivore species, such as the brown bear, that frequently need specific and urgent plans for conservation and management.

## Abstract (es)

La comunicación intraespecífica en los mamíferos está bien documentada, pero generalmente se limita a la señalización química y acústica. Sin embargo, otros canales ignorados, como la señalización visual, pueden ser utilizados para comunicarse entre conespecíficos. Aquí, utilizando manipulaciones experimentales junto con cámaras trampa en trece árboles de marcaje de oso pardo *Ursus arctos* en la Cordillera Cantábrica (noroeste de España), documentamos patrones temporales detallados y aspectos conductuales de un nuevo canal de comunicación recientemente descubierto para esta especie, es decir, la señalización visual a través del descortezado del tronco de árboles focales. Las imágenes de vídeo mostraron que el marcado visual es un medio de comunicación específico de sexo, edad y tiempo en los osos pardos, siendo realizado exclusivamente por los machos adultos durante la época de celo (principalmente entre abril y junio en el área de estudio). El descortezado del tronco siempre estuvo asociado al marcaje químico, y nunca fue un comportamiento aislado, lo que sugiere que las señales visuales y químicas podrían ser complementarias. Las marcas visuales pueden proporcionar una información diferente a las químicas, como por ejemplo el tamaño del individuo y, por tanto, el estatus de dominancia de los machos adultos en busca de oportunidades de apareamiento. Es la primera vez que se aportan pruebas que demuestran que la señalización visual en un gran carnívoro es exclusiva de una clase específica de individuos (los machos adultos) y está vinculada únicamente a las necesidades reproductivas. La señalización visual de los osos no sólo representa un avance en nuestra comprensión de la comunicación animal, sino que también puede servir para localizar fácilmente las zonas de celo de los mamíferos, lo cual es crucial para las especies de grandes carnívoros, como el oso pardo, que con frecuencia necesitan planes específicos y urgentes de conservación y gestión.

Animal communication is a well-studied topic for a large variety of taxa and can take several forms, for example, morphological, physiological, and/or behavioral (e.g., Vander Meer et al. 1998; Penteriani and Delgado 2017; Charlton et al. 2019). The communication process is fundamental to the persistence of the spatial and social structure within a population (Cornhill and Kerley 2020). At the individual level, a communication signal is perceived and interpreted by a receiver through sensory systems, that is, vision, hearing, touch, or olfaction (Gosling and McKay 1990; Rogers and Kaplan 2002). Among the different forms of mammal communication, chemical (e.g., Swaisgood et al. 2004; Campbell-Palmer and Rosell 2011; Morales-González et al. 2019) and acoustic (e.g., Chen and Wiens 2020) methods have been the most studied. One of the advantages of animal communication by scent-marking is that this form of signaling is long-lasting, and the information is available even in the absence of the sender (Cornhill and Kerley 2020). Actually, chemical signaling is expected to induce a change in the behavior of a receiver without any direct contact with the signaler, and with less energy expenditure by and/or risk to the sender than direct confrontations (Rogers and Kaplan 2002), thus benefitting both the sender and the receiver (Maynard Smith and Harper 1995).

Although research on mammal visual signaling is scarce and its role in communication has often been regarded as secondary, especially in crepuscular and nocturnal species, recent studies have shown that visual signaling could represent a more common and important form of mammal communication than previously thought (Caro et al. 2017; Penteriani and Delgado 2017; Negro et al. 2020; Penteriani et al. 2020, 2021). For example, long-lasting and permanent physical marks such as scratches and bites left by felines and ursids on diverse types of natural (e.g., tree trunks, rocks) and artificial (e.g., wooden poles) elements of the landscape have the potential to play an important role in animal communication (Cornhill and Kerley 2020; Penteriani et al. 2021).

Even though mammal scratches and bite marks have been reported since the 1930s (Green and Mattson 2003), they have been associated with claw sharpening (Green and Mattson 2003) or considered as an additional form of chemical communication via saliva deposition (Clapham et al. 2013; Taylor et al. 2015; Filipczyková et al. 2016; Gehring 2018; Cornhill and Kerley 2020). These marks have also been treated as incidental while performing chemical marking (Green and Mattson 2003). To our knowledge, the possibility that clawing and biting could represent some kind of visual signaling was first proposed by Burst and Pelton (1983) for the American black bear (*Ursus americanus*). This possibility was later supported by Thapar (1986) for tigers and Feldman (1994) for domestic cats, and more recently, Hirano et al. (2008) reported that mechanical marks made by howler monkeys (*Alouatta guariba clamitans*) could play the nonmutually exclusive function of being both a visual and chemical signal. To date, only one experiment (Penteriani et al. 2021) has been conducted to test the possibility that the marks produced by clawing and biting might represent a visual communication signal in a large carnivore, the brown bear (*U. arctos*), via tree debarking.

Brown bears are solitary, nonterritorial animals that use large areas leading to overlapping home ranges (Swenson et al. 2021). Providing information, for example, about age, sex, and reproductive status of an individual, through signals left in the environment such as chemical and visual marks (Maynard Smith and Harper 1995), is critical in the social interactions of solitary species because their encounter rates with conspecifics are much lower than in social species (Clapham et al. 2012; Lamb et al. 2017; Swenson et al. 2021). The more common communication behaviors in bears seem to be scent-marking via tree rubbing (where the back, neck, or shoulders leave secretions from sebaceous, and possibly apocrine glands located in the skin; Tomiyasu et al., 2018), urination, anogenital gland secretions, and/or pedal marking (Clapham et al. 2013, 2014; Lamb et al. 2017; Sergiel et al. 2017; González-Bernardo et al. 2021; Morehouse et al. 2021; Revilla et al. 2021). The suggestion of a new means of communication such as visual via physical marks on focal tree trunks (Fig. 1; Penteriani et al., 2021) has demonstrated that bear communication could be richer than generally believed and deserves further studies to better understand individual-related differences and temporal patterns of visual communication.

**Fig. 1.**
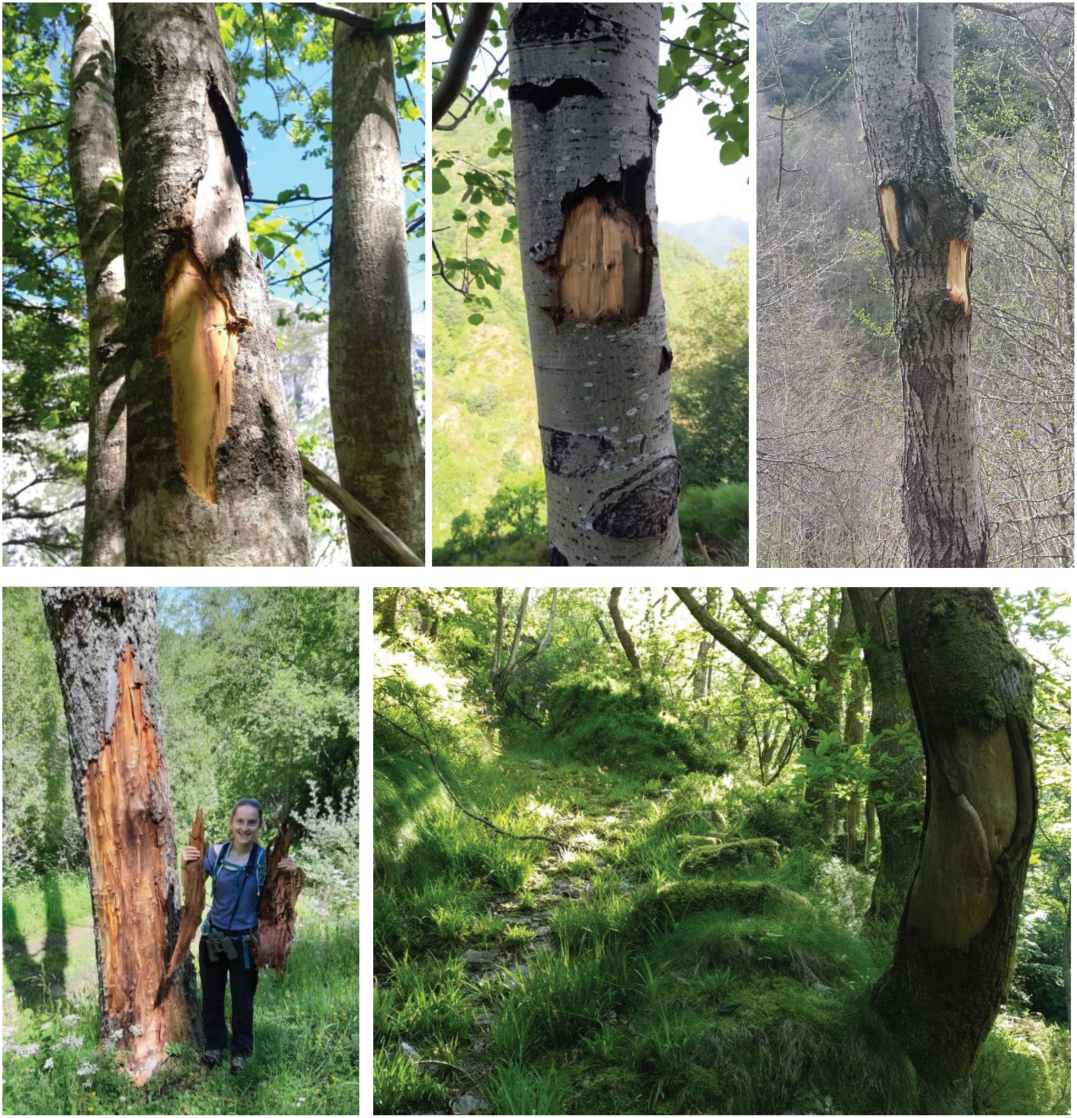
Examples of visual marks left by brown bear trunk debarking in the Cantabrian Mountains (northwestern Spain).

To this end, and following methods already employed in Penteriani et al. (2021) that presented the first evidence supporting the use of visual signaling by brown bears, we attempted to move our understanding of mammal visual communication a step forward by: (a) performing an experimental manipulation of bear tree marking behavior in the Cantabrian Mountains (northwestern Spain) during the 12 months of 2021; and (b) videorecording bear marking behaviors using camera traps. Because the previous research (Penteriani et al. 2021) was performed during the minimum time period (from the 1st of May to the end of September 2020) required to explore the possibility that brown bears might communicate by visual signals, this new study: (a) took into account the whole set of bear marking behaviors at rubbing trees throughout the entire year; (b) analyzed the entire set of bear sex and age classes interacting at rubbing trees; and (c) focused on yearly and daily temporal patterns of marking behavior. Additionally, while being conceptualized for visual marking, this study also allowed us to collect information on chemical communication behaviors–that is, it was not only possible to describe visual marking behavior throughout the year but also contextualize, describe, and compare visual signaling in relation to other bear marking behaviors at and around rubbing trees. If visual marking is (a) affected by sex and age, and (b) has a specific, season-dependent role in intraspecific communication, we expect adult males to display most of marking behaviors compared to lone adult females, females with cubs, and subadults during the mating season, mainly from early April to the end of June in our study area (Martínez Cano et al. 2016).

## Materials and Methods

### Study area.

**Fig. 2.**
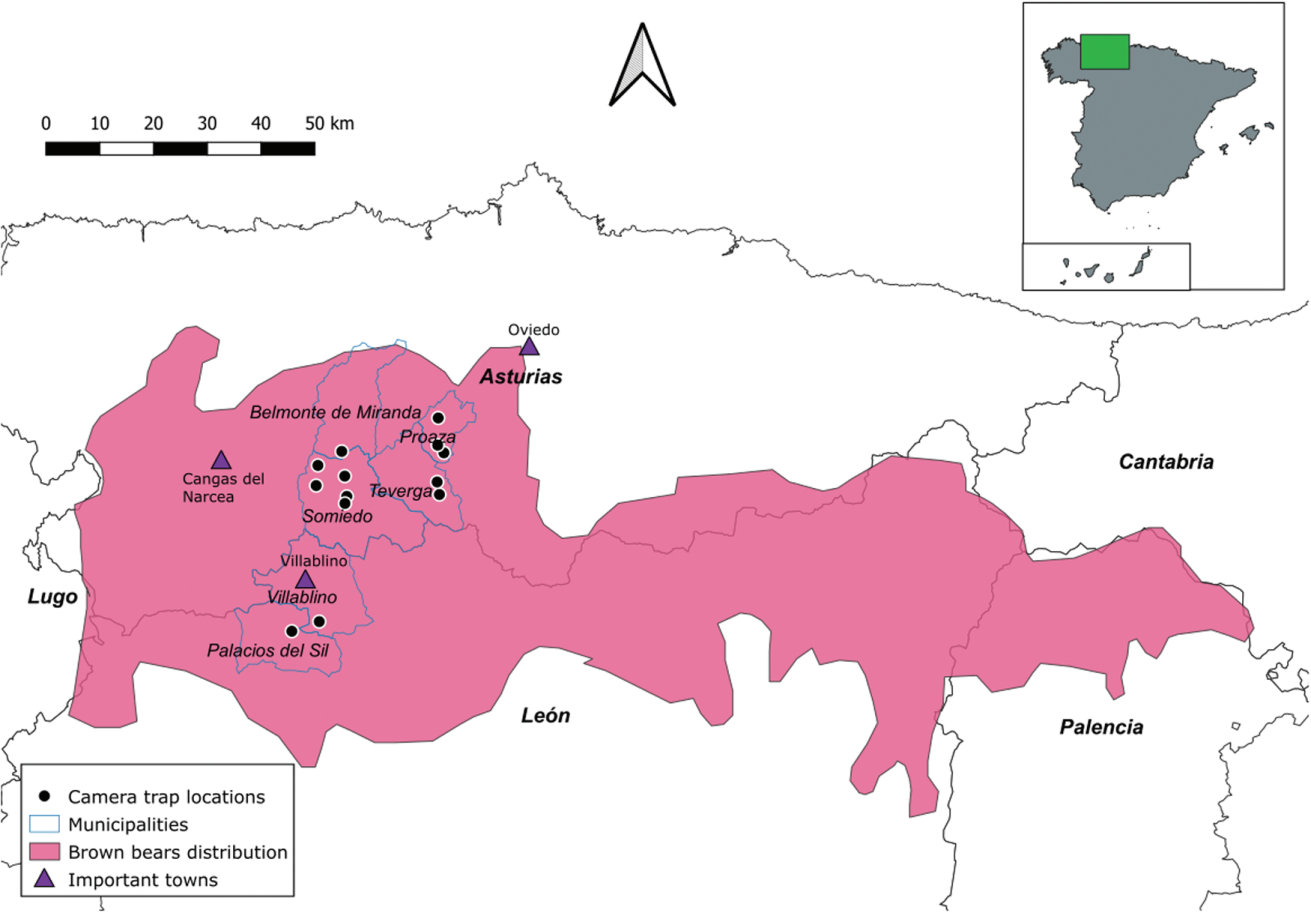
Locations of 13 camera traps (black dots) within the brown bear distribution area in the Cantabrian Mountains (northwestern Spain). Provinces (bold), municipalities (italic), and important towns (triangles) are also represented.

We conducted our study in the western Cantabrian Mountains (northwestern Spain) within the provinces of Asturias and León (Fig. 2). The temperate oceanic climate of the area is characterized by mild winters with no summer drought (Ortega and Morales 2015). The elevation ranges between 0 and 2,648 m above sea level (a.s.l.), with an average elevation of 1,100 m a.s.l. The landscape is mainly characterized by forests, shrubland, and pastures embedded in a matrix of crops, infrastructures, and human settlements (Zarzo-Arias et al. 2018). Forests are predominantly deciduous and mainly composed of oak (*Quercus petraea*, *Q. pyrenaica*, and *Q. rotundifolia*), beech (*Fagus sylvatica*), and chestnut (*Castanea sativa*) trees (Loidi 2017).

### Data collection and experimental protocol.

**Fig. 3.**
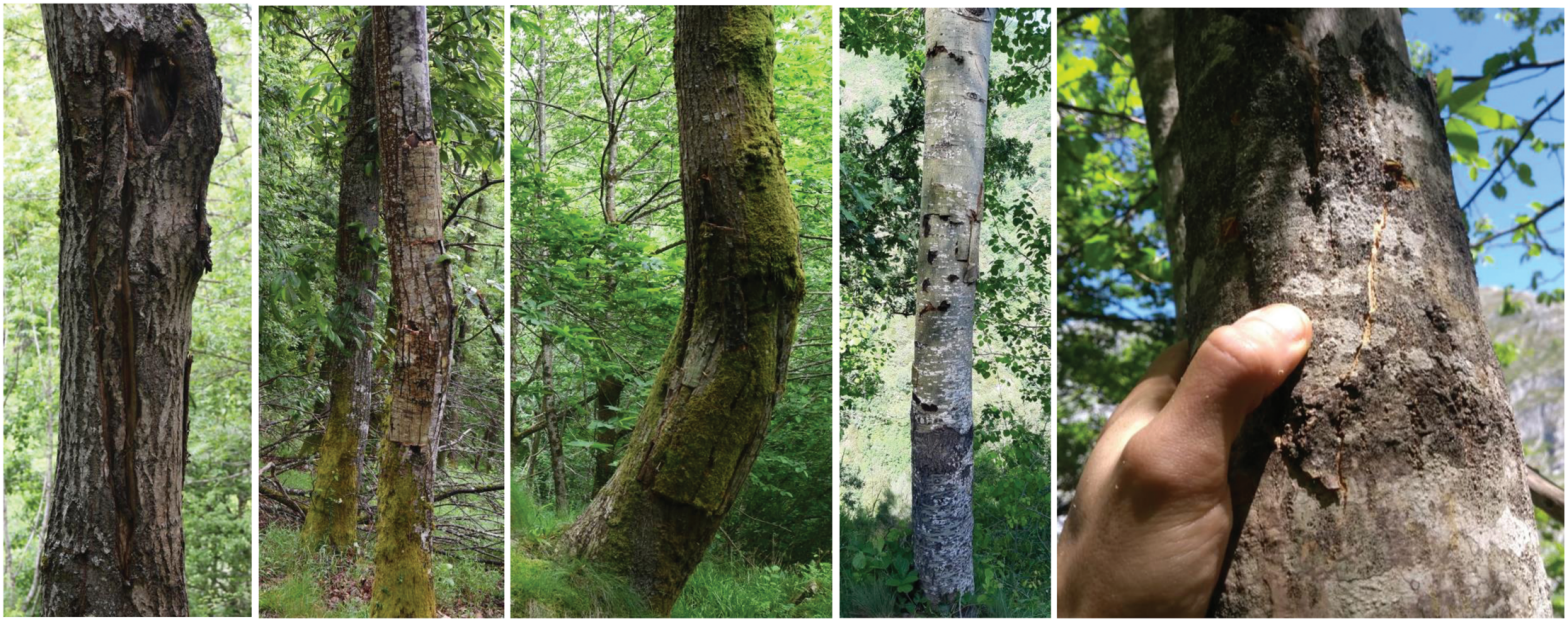
Examples of manipulations of brown bear visual marking. Marks made by bear claws and teeth were concealed with strips of bark from the same tree species, with the purpose of triggering a bear response to re-expose the concealed marks by removing the strips.

To describe brown bear marking behaviors we used a 2-fold approach. First, from 1 January to 31 December 2021 (for a total of 4,745 camera-trap days), we manipulated 13 already known marking trees used by bears (González-Bernardo et al. 2021): 11 in Asturias Province in the valleys of Pigüeña (Somiedo and Belmonte municipalities) and Trubia (Proaza and Teverga municipalities) and 2 in León Province in the valley of Sil (Villablino and Palacios del Sil municipalities; Fig. 2). Because hibernation is facultative in the Cantabrian Mountains, with multiple individuals not hibernating (Ruiz-Villar et al. 2019), we expected some marking behaviors to occur even during the hibernation period. Following the procedure of Penteriani et al. (2021), and to trigger the reaction of bears using preexisting marks on focal rubbing trees monitored by camera traps, we concealed the existing visual marks, one or more portions of the trunk where the bark has been removed (Fig. 1), with strips of bark from the same tree species (Fig. 3) at the end of December 2020. To conceal bear marks, we collected strips from the ground, or we debarked a distant (preferably recently dead) tree, to avoid any further interaction with the trees marked by bears. To avoid accidental removal of experimental bark by bears (e.g., bark loosely attached to the tree), we used nails to fix both the edge and the middle of each bark strip to the trunk (Penteriani et al. 2021). We installed one EREAGLE E3 Trail Camera (http://www.ereagle.com/Index.asp) with infrared function at each of the 13 brown bear marking trees, approximately 3–5 m from the focal tree. We did not use bait to attract bears but rather relied solely on the spontaneous marking activity of local bears. The cameras were set to record 60-s videos with a 1-s delay. Each video included the date and time. Sites were visited once a month during the study period to service the camera traps and, eventually, reconceal a bear mark in the case that it was debarked. Additionally, in order to have more material to describe visual marking behavior, we also used opportunistically collected photos (*n* = 79 photos, corresponding to 10 independent events) and videos (*n* = 43 videos, corresponding to 36 independent events) from 2008 to 2020 from six camera traps (i.e., one camera running from 2008 to 2020, one in 2019, and four in 2020) in the same valleys where the manipulative experiment was performed (Fig. 2).

Our research followed ASM guidelines (Sikes et al. 2016) and was approved by the Principality of Asturias (AUTO/2021/172) and the Junta of Castilla and León (AUES/CYL/248/2021).

### Type of behaviors.

We determined a behavioral sequence each time a bear triggered a camera. The sex of individuals was determined from external reproductive organs, swollen mammae, the presence of cubs, or any distinctive sign of the identity of an individual when a previously known bear (Penteriani V., National Museum of Natural Sciences, Madrid, Spain; Hartasánchez A., FAPAS, Ausurius, Spain; December 2022) used the focal tree. Individuals were grouped into seven classes: adult male, adult female, unknown adult, female with cubs, male subadult, female subadult, and unknown subadult. Specific characteristics of individuals, such as fur marks, color, and body morphology, as well as previously collected camera-trapping data on focal individuals (Zarzo-Arias et al. 2018) also allowed for the characterization of bear sex and ages. In particular, a bear was identified as a subadult on the basis of its morphology (e.g., the ears appear close together and disproportionately large compared to the rest of head, the head, neck, and shoulders are elongated; the neck appears long and thin; and the legs appear long compared to the body) and its size when compared with other bears frequenting the same rubbing tree.

Behavioral analyses were performed using the free software BORIS 7.10.1 (https://www.boris.unito.it/; Friard and Gamba 2016). This software was used to record and quantify the time spent performing each behavior present beforehand in a defined ethogram. Using existing ethograms from previous studies (Taylor et al. 2015; Zarzo-Arias et al. 2018), we assigned brown bear marking behaviors at trees into five categories: (1) dorsal marking, where the bear rubs the tree with its back; (2) facial marking, where the bear rubs the tree with its head and/or neck while facing the tree; (3) pedal marking, where the bear creates ground depressions by stepping and twisting its feet on the terrain surrounding rubbing trees; (4) tree olfactory investigation (i.e., sniffing), where the bear visibly smells the tree or the surroundings of the tree; and (5) visual marking, where the bear removes bark (both natural or manipulated) with its claws and/or teeth. For most analyses, natural and experimental debarking were considered as one behavior as our focus was on the use of visual communication regardless of the means, that is, tree debarking to produce a new, fresh mark, or debarking of an experimental concealed mark, except for the time spent making a new mark, which was longer than that necessary to just remove strips concealing a manipulated mark. The premise that a bear is communicating visually by removing manipulated bark is based on the results of our previous experiment (Penteriani et al. 2021), where only bark strips experimentally covering bear marks were removed during the experiment. Actually, control bark strips fixed to (a) the same trunk as the manipulated bear mark, (b) the nearest neighboring tree to the manipulated one showing bear marks, and (c) the nearest rubbing trees with no bear marks were never removed by bears. Finally, we recorded the height of the top of the visual marks on the tree trunks, the highest point reached by the bear when debarking was classified as ‘above the shoulder line’ or ‘below the shoulder line’ of a bear. The purpose of this measurement was to determine whether most of the marks were made at the highest point a bear could reach with its mouth or paws when standing up. If visual marks represent the highest point a bear can reach, they should be made on a portion of the trunk where chemical signals via dorsal/head rubbing are difficult to leave and, thus, visual signaling might have a different purpose in bear communication.

### Behavioral sequences of marking.

Each marking activity was included in a behavioral sequence to determine the probabilities of transition between diverse behaviors when visual marking was performed. To describe the whole behavioral sequence, we analyzed bear behaviors from the first behavior performed at the marking tree until the departure of the bear. We built a behavioral sequence diagram to represent the linkage and the order of the diverse marking behaviors by constructing a first-order Markov chain to obtain the probabilities of transition between the different recorded behaviors. A Markov chain is a stochastic process to measure probabilities, following the Markov property. It means that future behaviors, represented as states, depend only on the present state and are independent of past states (Van Kampen 2007). The probabilities associated with various state changes, moving from one state to another, are transition probabilities, represented in a transition matrix (Clapham et al. 2014) provided by BORIS. This allowed us to identify relationships between the different behaviors. Transitions with a probability inferior to 0.1 were removed from the analysis to avoid low sample sizes and to focus on the predominant transitions only (Clapham et al. 2014). For this analysis, dorsal and facial marking have been coupled as one single behavior for the ease of reading and because the information provided by those two behaviors is presumed to be the same (i.e., chemical signaling).

### Statistical analysis.

To examine frequencies and lengths of each of the marking behaviors included in our ethogram, we calculated: (1) relative proportions of recorded behaviors; (2) proportions per sex and age class; and (3) proportion of time spent doing each behavior. For this last variable, only natural visual marking recorded by videos from 2015 to 2020 (*n* = 7) were included in the analysis, where debarking could actually be timed, as experimental debarking was often very fast and not representative of the effort put into creating a new visual mark. To examine if there was a significant difference in time, we performed a one-way ANOVA followed by a Tukey post hoc test.

Temporal patterns of marking activity were analyzed at monthly and daily scales. We used the relative independent capture (RIC = *independent captures of the month/camera-trap days of the month* × 1,000; Zahoor et al. 2021) to compare activity among months. To account for day length variation, that is, 6-h difference in luminosity duration with on average 9h21 of light in January, against 15h18 in June, we considered time in two different manners. First, we built four time periods (e.g., Hanya et al. 2018; Zahoor et al. 2021): sunrise (1 h before and 1 h after official sunrise time); day (from 1 h after sunrise to 1 h before the sunset); sunset (1 h before and 1 h after official sunset time); and night (from 1 h after sunset to 1 h before sunrise). This approach accounts for exact day length as a function of seasonal variations. Secondly, we converted clock hours to a continuous single number between −1 and +1, considering both sunrise and sunset.

To examine temporal variation in marking activity, we conducted two kinds of analysis. First, we performed χ² goodness-of-fit tests over the entirety of the marking events videorecorded by camera traps. We then performed post hoc tests (binomial exact tests) with Bonferroni correction. We tested whether the marking activity was significantly different between the 12 months of the study (monthly pattern) and among the four daily periods (circadian pattern; sunrise, day, sunset, night), by comparing the observed and expected values corrected for the variation in days between months and the difference in length between the daily periods. Second, to examine the daily and monthly variation in probability of making a visual mark, we constructed a generalized linear model with a binomial family. We used debarking (yes/no) as the response variable, and Julian date and time (a number considering both sunrise and sunset) as explanatory variables. Because of the small sample size, tree identity (*n* = 13) as a random factor could not be included in the model. All analyses were carried out using R.3.6.1 (R Development Core Team 2018).

## Results

### General patterns and description of marking behavior at rubbing trees.

Over our systematic, year-long study, we videorecorded 263 independent events of brown bear visits to rubbing trees, including 239 marking or investigation events. In addition to these events, 46 opportunistic events from 2008 to 2020 were also available (10 sets of pictures and 36 sets of videos), for a total of 309 brown bear behavioral events at rubbing trees.

**Fig. 4.**
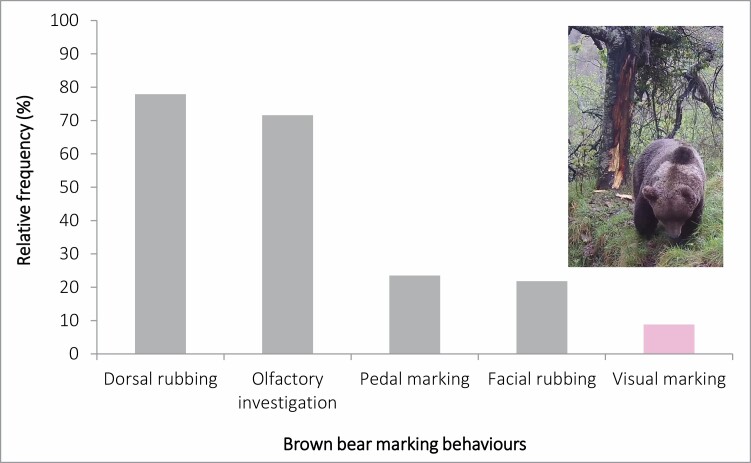
Relative frequencies of the different types of brown bear marking behaviors, including dorsal marking, investigation, pedal marking, facial marking, and visual marking, collected during 46 opportunistic (2008–2020) and 239 systematic (2021) observations by camera traps placed at rubbing trees in the Cantabrian Mountains (northwestern Spain).

Dorsal rubbing was the most frequent behavior performed by brown bears, displayed at 77.9% of all visit events (*n* = 222). Dorsal rubbing was followed by olfactory investigation (71.6%, *n* = 204), pedal marking (23.5%, *n* = 67), facial rubbing (21.8%, *n* = 62), and visual marking (8.8%, *n* = 25; Fig. 4). When taking into account debarking to produce a new visual mark only, with debarking of manipulated marks excluded, clawing and biting were performed during 11 and 8 events, respectively.

Out of the 285 recorded events (the 239 events from the systematic, year-long study + the 46 opportunistic events), we were able to accurately identify the age and sex of individuals in 248 events, which showed variations in the different classes of behaviors performed according to the age and sex of individuals (Table 1). Olfactory investigation alone was performed by all classes, with a majority occurring in subadults (Table 1). Adult males represented the age class that interacted the most with rubbing trees and their surroundings, and the only one that performed all types of behaviors. In fact, adult males: (a) rubbed and investigated the most compared to other behaviors, with 89 events against 14 for females, 38 for subadults, 3 for cubs, and 14 for unknown individuals; (b) marked the most without any prior olfactory investigation, with 44 events against 1 for females and 8 for unknown individuals; and (c) predominantly performed pedal marking. Furthermore, and central in the context of visual communication, (d) visual marking appeared to be only performed by males (100% of the events; Table 1, Supplementary Data SD1). In two cases, not considered in the analyses, a cub and a subadult removed our experimental marks unintentionally, as a consequence of rubbing against the tree or holding onto it. The proportion of time used for each behavior (Table 2) was slightly different (*F*_5,36_ = 2.68, *P* = 0.04). In particular, Tukey post hoc tests revealed marginal differences only between the time a bear performed (a) dorsal marking and biting (*t* = 3.05, *P* = 0.05), and (b) dorsal and pedal marking (*t* = −3.08, *P* = 0.04).

**Table 1.** Age and sex classes of brown bear marking behaviors (shown as %; see Materials and Methods for more information on the bear ethogram) at rubbing trees, recorded by camera traps during 46 opportunistic (2008–2020) and 239 experimental (2021) observations in the Cantabrian Mountains (northwestern Spain).

Sex/age class	Investigation	Investigation and marking	Marking	Visual marking	Pedal marking
Adult male	24.4	56.3	70.1	100.0	90.8
Adult female	15.6	8.9	9.1	0.0	0.0
Subadult	37.8	24.1	2.6	0.0	3.1
Cub	4.4	1.9	2.6	0.0	0.0
Unknown	17.8	8.9	15.6	0.0	6.2

**Table 2.** Mean ± standard deviation (*SD*), minimum (Min), and maximum (Max) amount of time (in seconds) of each type of brown bear marking behavior at rubbing trees when visual marking with claws and teeth occurred (*n* = 7). Videorecorded information was collected by camera traps in the Cantabrian Mountains (northwestern Spain) between 2015 and 2020. Only bears making new visual marks were included in the analysis, where debarking could actually be timed, as experimental debarking was often very fast and not representative of the effort put into creating a new visual mark.

Behavior	Mean ± *SD*	Min–Max
Dorsal marking	28.1 ± 16.2	0–49.2
Clawing	12.5 ± 8.5	0–24.9
Olfactory investigation	11.7 ± 13.8	0–37.4
Facial marking	10.6 ± 13.9	0–35.1
Biting	8.5 ± 7.2	0–16.6
Pedal marking	8.3 ± 9.6	0–26.2

### Characteristics of visual communication.

We were able to determine the height of 24 visual marks, 62.5% being located above the shoulder line of the marking individual, where the probability of marking chemically using the back and the neck is low or null (Supplementary Data SD1). Only two of these marks extended far enough down the trunk to be accessible for chemical marking as well. Furthermore, during the peak of marking activity, April and May, most of the marks (74%, *n* = 19) were located above the shoulder line. In contrast, 37.5% of visual marks were located below the shoulder line, with one mark at the very base of the tree. Those marks located below the shoulder line occurred most frequently (80% of the marks, *n* = 5) when visual marking was less intense, at the very beginning (March) and at the end (June and July) of the mating period.

**Fig. 5.**
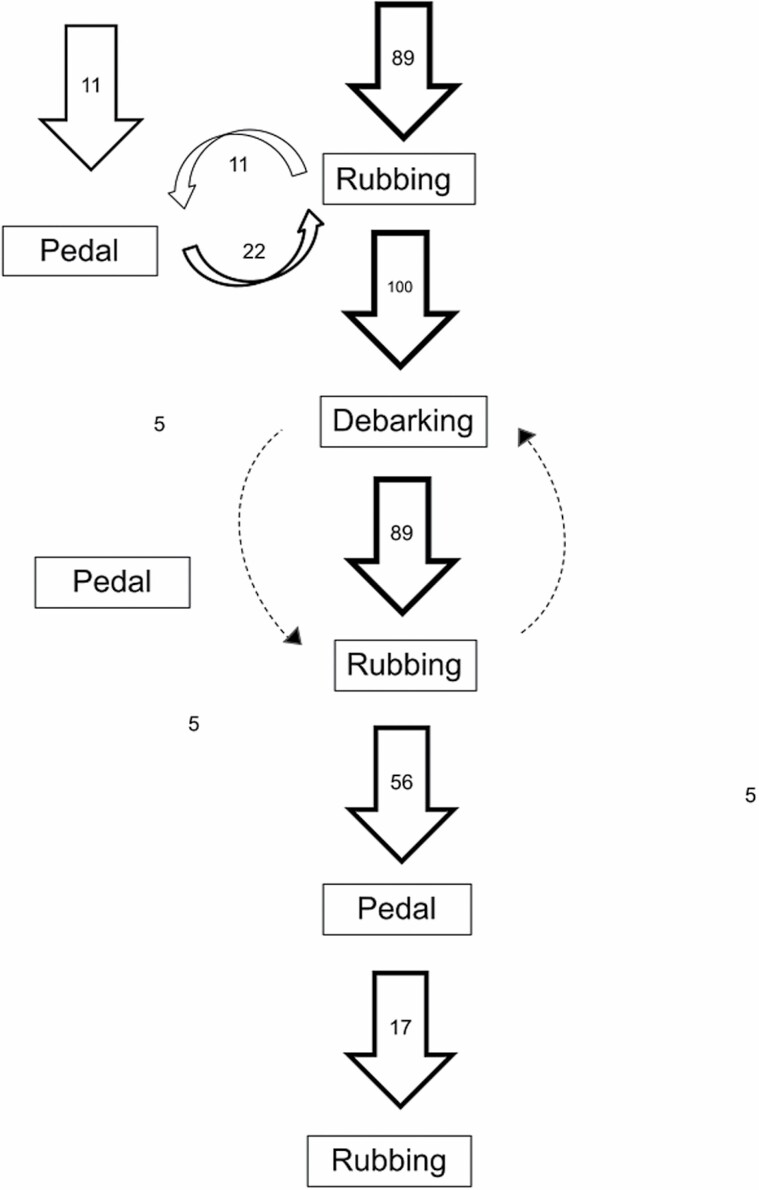
Behavioral sequence diagram of adult male brown bears at marking trees. Behaviors include pedal marking (Pedal), face and/or back rubbing (Rubbing), and visual marking including clawing, biting, and removing of our experimental bark strips (Debarking). The behavior “investigation” at the focal tree has been removed from the diagram because of its occurrence at every transition and because investigation had high and similar probabilities of transition with every other behavior in the sequence (Fig. 6). Numbers represent the transition probabilities between postures. Dotted arrows indicate reiterative transitions between debarking and rubbing. Data are an assemblage of opportunistic observations from 2015 to 2020 (*n* = 9), and experimental data from 2021 (*n* = 10) from camera traps placed at rubbing trees in the Cantabrian Mountains (northwestern Spain).

**Fig. 6.**
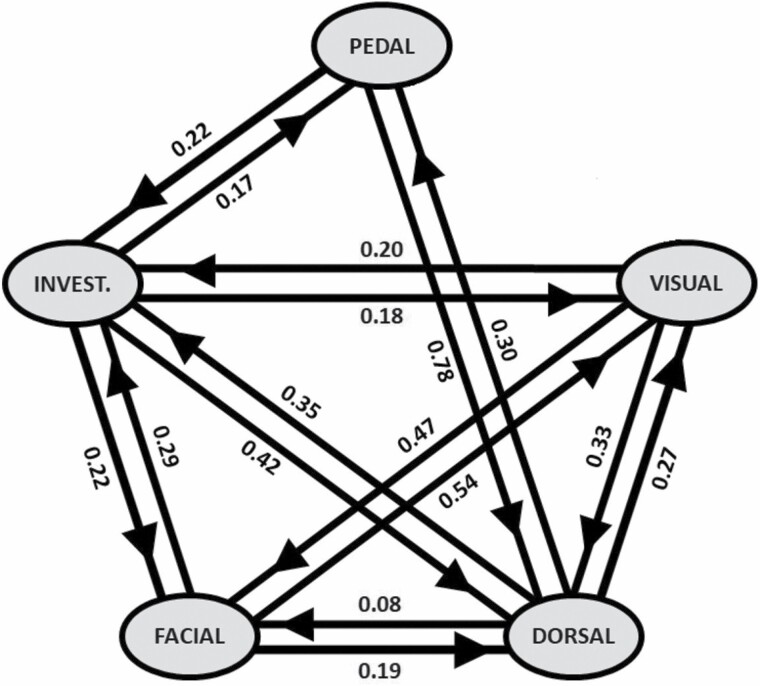
Markov chain diagram of adult male brown bears displaying behavioral sequences at marking trees. Behaviors include pedal marking (Pedal), investigation (Invest.), facial marking (Facial), dorsal marking (Dorsal), and visual marking including clawing, biting, and removing our experimental mark (Visual). Data are an assemblage of opportunistic observations from 2015 to 2020 (*n* = 9), as well as experimental data from 2021 (*n* = 10) from camera traps in the Cantabrian Mountains (northwestern Spain).

Nineteen visual marking videos out of 309 recorded behavioral events revealed that the behavioral chain displays a clear pattern in behavioral sequence, with rubbing as the entry behavior (Fig. 5). During 89% of the events, individuals interacted with the tree by first rubbing either their back or their face on the tree (Fig. 5). Actually, dorsal marking was linked to all the other behaviors, which converged to dorsal marking with a high probability of transition (Fig. 6). In addition to being the entry behavior, dorsal rubbing also represented an important exit behavior in the behavioral sequence. Pedal marking mainly occurred at the beginning or at the end of the sequence (Fig. 5) and it was only linked with dorsal marking and investigation (Fig. 6). Finally, visual marking was always performed after chemical marking (100%, *n* = 19) and most of the time it was followed by a second round of chemical marking (95%, *n* = 18; Fig. 5). Additionally, visual marking was: (a) also linked with investigation, dorsal, and facial marking (Fig. 6); and (b) mainly preceded and followed by facial marking (54% and 47%, respectively), dorsal marking (27% and 33%, respectively), and investigation (19% and 20%, respectively; Fig. 6). Unlike rubbing and investigation, which can also be performed alone, that is, not within a behavioral sequence, claw and bite marking was always included in a chemical marking sequence and never performed alone. Pedal marking was mostly associated with a chemical sequence, even if it was performed alone twice and associated with investigation six times.

Except at one rubbing tree, where the same adult male removed experimental strips twice (8 March and 16 June), debarking only occurred once per rubbing tree per year, even when other adult males frequented and/or chemically marked the rubbing tree.

### Temporal patterns of marking behavior.

**Fig. 7.**
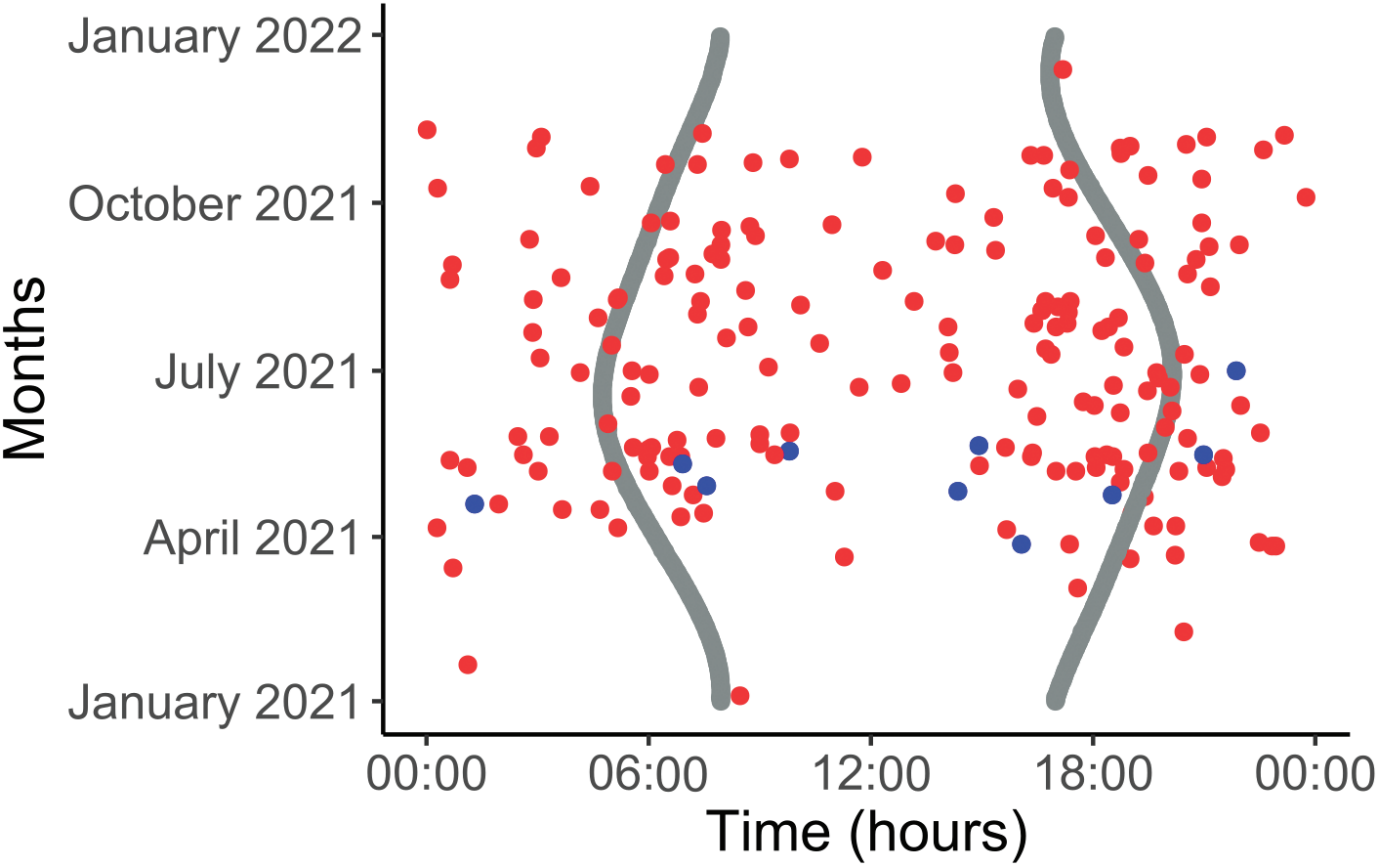
Actogram of brown bear marking activity in the Cantabrians Mountains (*n* = 196). The *x*-axis indicates the hours of the day (from 0 to 24 UTC, coordinated universal time). The *y*-axis indicates the days of the year, from 1 January to 31 December 2021. Continuous gray lines indicate the time of sunrise and sunset. Light and dark circles represent chemical and visual marking, respectively.

**Fig. 8.**
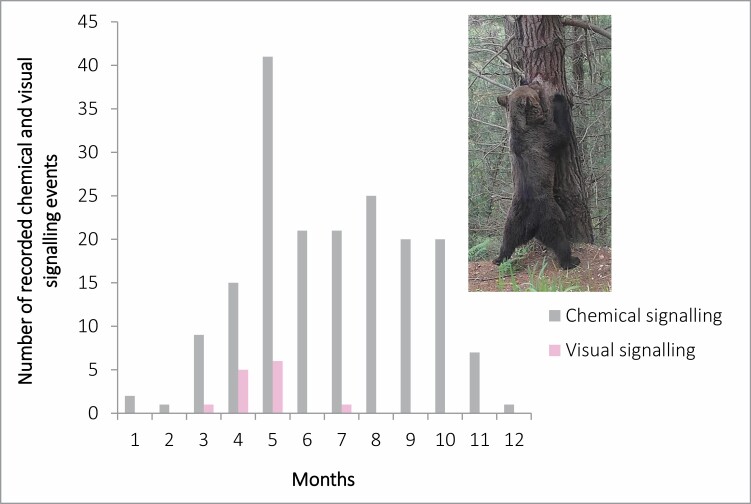
Monthly marking activity of brown bears at rubbing trees (*n* = 196 records), recorded by camera traps in 2021 in the Cantabrian Mountains (northwestern Spain).

Bears showed variable marking activity throughout the entire 24-h-day period during which they were active (Fig. 7). Marking behavior as described by the RIC differed significantly among the 12 months of the study (χ^2^ = 113.8, d.f. = 11, *P* < 0.001), lowest in January, February, and December (*P*_Jan_ < 0.001; *P*_Feb_ < 0.001; *P*_Dec_ < 0.001), mostly during the hibernation period. Thereafter, marking activity gradually increased to reach a peak in May, during the peak of the mating season, where marking activity was significantly higher than expected (*P*_May_ < 0.001; Fig. 8). Visual signaling activity by claw and bite marking followed the same trend as that of chemical marking but could not be tested statistically because of its reduced sample size. Nonetheless, we can conclude that visual signaling was completely absent during the winter, as this behavior was exclusively performed during the mating season, reaching its peak synchronously with chemical marking in May (Fig. 8).

**Fig. 9.**
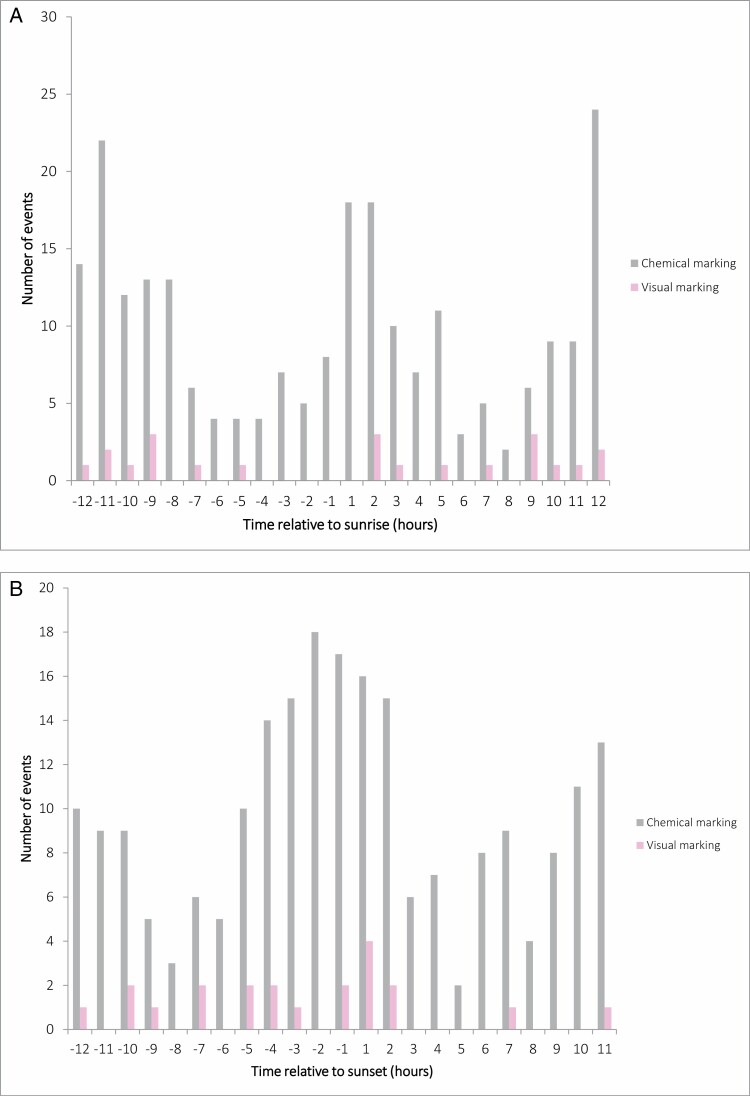
Temporal distribution of 234 brown bear chemical and visual marking behaviors at rubbing trees in the Cantabrian Mountains (northwestern Spain), represented as time relative to (A) sunrise and (B) sunset.

The marking activity was significantly different during the day (χ^2^ = 20.8, d.f. = 3, *P* = 0.0001), with its frequency being higher than expected (*P* = 0.011) at sunset and lower than expected (*P* = 0.0001) at night. The peak of activity slightly before sunset and at sunset was complemented by another more diffuse peak in the morning, after sunrise (Fig. 9A and B). In particular, even if visual marking can occur during the 24-day period, it is more frequent during the daytime, and reaches a small peak around the sunset period (Fig. 9B). When testing whether time and month have an influence on the probability of making a visual mark, Julian date had an effect on the probability that bears make visual marks on trees (Table 3). Additionally, both time and the interaction between time and Julian date were included in the competing models (ΔAICc < 2), meaning that the hour of the day may explain some of the variance recorded for the temporal occurrence of debarking (Table 3).

**Table 3.** Summary of the generalized linear model outputs of the factors potentially affecting the probability of male brown bears making visual marks in the Cantabrian Mountains (northwestern Spain) in 2021 (*n* = 236 behavioral events).

Variable	Estimate	Lower CI (2.5%)	Upper CI (97.5%)	*SE*	*Z*	*P*
Intercept	–0.83	–2.6	0.98	0.92	0.90	0.37
Time	1.44	–1.60	4.49	1.55	0.93	0.35
Julian date	–0.02	–0.03	–0.004	0.006	2.60	0.01
Time: Julian date	–0.01	–0.04	0.01	0.01	1.10	27

## Discussion

Our results confirmed that remote communication in brown bears is dominated by chemical marking (Clapham et al. 2012; Tattoni et al. 2015; Lamb et al. 2017; Revilla et al. 2021), with dorsal rubbing being the predominant behavior, together with additional behaviors leaving chemical markers, including facial rubbing and pedal marking. The general temporal patterns of chemical marking recorded in the Cantabrian brown bear, with low marking activity in January and February and a peak during the mating season (April–June; Fig. 8), support previous findings in North American and Russian brown bear populations, in which chemical communication is performed during both mating and nonmating seasons, principally by males (e.g., Green and Mattson 2003; Clapham et al. 2012, 2014; Seryodkin 2014; Lamb et al. 2017).

More noteworthy, our study seems to confirm the importance of visual marking in a large carnivore, namely the brown bear, yielding new insight into its patterns and characteristics. Trunk debarking is well integrated into typical brown bear communication sequences and, in our opinion, may provide additional information that is not available through chemical marking on the characteristics of marking individuals (adult males) during the mating period. The sex- and time-specificity of brown bear visual marking determines that its frequency was the lowest of all the other types of marking behavior, which can explain the results of previous studies regarding the general marking behavior of brown bears (Green and Mattson 2003; Taylor et al. 2015; González-Bernardo et al. 2021; Tattoni et al. 2021). This also means, in our opinion, that visual signaling should not be considered a secondary communication channel because of its low frequency during the year (Clapham et al. 2013)–more properly, this behavior is a sex- and age-specific means of communication with a precise purpose that plays a role in a narrow temporal window. The highlighted restricted temporal range of brown bear visual signaling (mating period only) and its use by a single bear class (adult males) made our sample size relatively small. However, and also considering the many bear interactions recorded at rubbing trees during the entire year, we are confident that the small number of bears performing this behavior is mainly attributable to the high specific context of visual signaling.

As a general rule, brown bear marking behavior was also dependent on sex and age classes. Indeed, rubbing was a behavior performed by all categories of bear but highly dominated by males, as was found in previous studies on brown bears (e.g., Clapham et al. 2014; Revilla et al. 2021; Tattoni et al. 2021) and felids (Allen et al. 2015; Kusler et al. 2019; Cornhill and Kerley 2020). Both investigating and rubbing were mainly dominated by males, even if also performed, at a low frequency, by all sex and age classes (see also Tattoni et al. 2015, 2021). Investigation, which allows individuals to obtain information about the presence of other individuals, was performed by all individuals (see also Tattoni et al. 2015; Revilla et al. 2021), mainly subadults, followed by females and males at an equal frequency. That is, the information left on the rubbing tree is received by all types of bears, irrespective of their age or sex (see also Clapham et al. 2012; Revilla et al. 2021).

These findings reinforce the idea that remote hierarchy is transmitted via scent-marking (Lamb et al. 2017; Revilla et al. 2021). Indeed, dominant males will actively and repeatedly scent-mark to communicate their presence and obtain information, whereas subadults will investigate more than they mark to obtain information about the dominant males in the surrounding area and engage in avoidance behaviors (Jojola et al. 2012). Females may investigate for two main reasons. First, if the female is with cubs, she may investigate to avoid potential males present in the area and, thus, reduce the risk of infanticide (Swenson et al. 2021). Second, lone females can probably use this olfactory information to determine the presence and identity of males during the mating season, which may influence their choices regarding offspring paternity (Morehouse et al. 2021). Finally, both visual and pedal marking seemed to have similar patterns, such that these two behaviors appear to be quasi-exclusively (pedal marking; Revilla et al. 2021) and exclusively (tree debarking) performed by males during the mating season.

This clear male-performance pattern leads to the hypothesis that visual marking gives mating-related information. Males could indeed use this type of marking mainly to provide information about their size to females and/or subordinate males (e.g., other adult males and subadults sharing the same area), information that does not seem possible to convey by chemical signaling. In our opinion, this possibility is supported by the height at which visual marks are made (see also Karamanlidis et al. 2007; Seryodkin 2014), which are higher on the trunk during the peak of the mating season (April and May). During the most active mating period, visual marking could be used by males to inform other conspecifics sharing the same mating areas about their size and, thus, their dominance status to avoid/reduce risky conflicts, as physical injuries are typical of male fights during mating (Swenson et al. 2021). The combination of a visual mark above chemical information might allow bears to convey diverse and complementary information about themselves, for example, presence, identity, sex, and size in the same communication spot (the rubbing tree). However, we also observe that some marks are made closer to the tree base (e.g., when the mark is made by a bear in a four-legged position), which would be confounding if the only function of debarking was to provide information about the size of the individual.

Before our experimental approaches (this study and Penteriani et al. 2021) supported the visual signaling function of brown bear debarking, diverse hypotheses had been suggested concerning the function of bear claw and teeth marks. Even if some purposes might not be exclusive to visual communication, their role (if any) has not been identified. Although we do not know whether certain exclusive compounds may be found in saliva (or in saliva during the mating season only), the quantity of secretion through salivation may be low compared with that left while rubbing. As described earlier, visual marking is always coupled with chemical marking and those behaviors, especially back rubbing, should leave a higher concentration of chemical information than that left by biting trees. Additionally, when bears removed experimental bark strips concealing their marks, the duration of the interaction of their claws and teeth with the trunk lasted for only a few seconds, resulting in a low quantity of secretions left on the tree. Furthermore, none of the collected videos support the idea that bears bite trees to feed on the bark, and, if the purpose of the mark was feeding, all bear ages and sexes would likely take advantage of this food source, not only adult males during a very limited period of time. Finally, and without denying the possibility that clawing may also leave secretions from pedal glands on trees (e.g., Clapham et al., 2013), the new information recently revealed about pedal marking and its chemical transmission efficiency (Sergiel et al. 2017; Revilla et al. 2021), coupled with our results regarding its frequency (68% of our visual marking sequences included pedal marking), does not support the idea that the primary objective of clawing is to leave secretion from pedal glands. Finally, we would like to stress here that, even though we did not wear gloves when handling experimental bark, if bears were just detecting our scent and reacting to it, they would have removed experimental bark throughout the year, not just when they were presumed to use visual marks to communicate during the mating period. Additionally, if bears were attracted by our scent: (a) not only males during the mating period would have been attracted; and (b) the dozens of control bark strips we used in Penteriani et al. (2021) would also have attracted bears. Instead, we consider that the likelihood of being attracted by the smell of control bark was higher than that of being attracted by bark covering bear marks because for each covered bear mark there were three control bark strips (Penteriani et al. 2021) fixed on: (1) the same trunk as the manipulated bear mark; (2) the nearest neighboring tree to the manipulated one showing bear marks; and (3) the nearest rubbing trees with no bear marks.

The behavioral sequence confirmed visual marking as an integral part of the communication behavior sequence, paired and in association with chemical marking. The marking behavioral sequence at rubbing trees is relatively simple, with a succession of scent and visual marking, in accordance with the previous findings of Clapham et al. (2014). Some sequences were more complicated, with additional pedal marking, rubbing, and iterative transitions during the sequence. Furthermore, the Markov chain helps us determine hotspot behaviors. Investigation and dorsal marking appear to be fundamental and central to the sequence, being linked with a high probability to every behavior in the sequence, while pedal marking and visual marking seem to be more specific and constrained to certain parts of the sequence. Because it seems that only one adult male per year leaves visual marks on the same rubbing tree, even when the latter is a marking spot for several individuals, we can hypothesize that males that visually mark might be the locally dominant individuals and have higher female recruitment. Future studies should clarify the potential link between the identity of males visually marking and their reproductive performance in terms of the number of mates, for example, especially in comparison with other adult males sharing the same area.

As per the daily marking activity pattern, marking can occur both during the day and at night; however, bear marking behavior tends to be more frequent during daylight hours with a decrease in the middle of the day and a peak at sunset, followed by a second peak in the morning around sunrise (Fig. 9). This distribution of marking activity is coincident with brown bear activity rhythms in the Cantabrians Mountains (Vazquez Garcia 2020), where bears show: (a) a peak of activity around sunset; (b) a decrease in activity at night and in the middle of the day; and (c) a weaker peak in the morning.

To conclude, both spontaneous visual marking and bear reactions triggered by our experimental manipulations of trunk marks suggest that visual communication is an important channel for male bears during the mating season. This communication channel seems to be associated with and complementary to the chemical channel and could provide supplementary information. These findings, which help to more thoroughly understand brown bear and, more generally, mammal communication behavior via visual signaling, also have the potential to represent an important tool in the conservation and management of large carnivores. In fact, because ursids and felids, at a minimum, have been reported to use visual signs to communicate, and these species and/or some of their populations have conservation concerns, the conspicuousness of visual marks to humans can help to quickly and easily detect the main reproductive areas of large carnivores. This represents crucial information in landscape planning when people and large carnivores share the same environment, as is increasingly common in human-modified landscapes (Morales-González et al. 2020).

## Supplementary Material

gyac126_suppl_Supplementary_Data

## Supplementary Data

**Supplementary Data SD1.**—Video footage of brown bear trunk debarking for visual signaling purposes, as recorded by camera traps placed in front of rubbing trees in the Cantabrian Mountains. The first three sequences correspond to bears making fresh marks on rubbing trees: same tree during 2018 and 2019 (two diurnal sequences), and the first nocturnal sequence from 2020. The following four sequences are from bears removing bark strips used in the experimental approach of this study (2021).
